# Dopamine Transporter Loss in 6-OHDA Parkinson’s Model Is Unmet by Parallel Reduction in Dopamine Uptake

**DOI:** 10.1371/journal.pone.0052322

**Published:** 2012-12-26

**Authors:** Tanya Chotibut, Deana M. Apple, Rebecca Jefferis, Michael F. Salvatore

**Affiliations:** Department of Pharmacology, Toxicology, and Neuroscience, Louisiana State University Health Sciences Center, Shreveport, Louisiana, United States of America; Karolinska Inst, Sweden

## Abstract

The dopamine transporter (DAT) regulates synaptic dopamine (DA) in striatum and modulation of DAT can affect locomotor activity. Thus, in Parkinson’s disease (PD), DAT loss could affect DA clearance and locomotor activity. The locomotor benefits of L-DOPA may be mediated by transport through monoamine transporters and conversion to DA. However, its impact upon DA reuptake is unknown and may modulate synaptic DA. Using the unilateral 6-OHDA rat PD model, we examined [^3^H]DA uptake dynamics in relation to striatal DAT and tyrosine hydroxylase (TH) protein loss compared with contralateral intact striatum. Despite >70% striatal DAT loss, DA uptake decreased only ∼25% and increased as DAT loss approached 99%. As other monoamine transporters can transport DA, we determined if norepinephrine (NE) and serotonin (5-HT) differentially modulated DA uptake in lesioned striatum. Unlabeled DA, NE, and 5-HT were used, at a concentration that differentially inhibited DA uptake in intact striatum, to compete against [^3^H]DA uptake. In 6-OHDA lesioned striatum, DA was less effective, whereas NE was more effective, at inhibiting [^3^H]DA uptake. Furthermore, norepinephrine transporter (NET) protein levels increased and desipramine was ∼two-fold more effective at inhibiting NE uptake. Serotonin inhibited [^3^H]DA uptake, but without significant difference between lesioned and contralateral striatum. L-DOPA inhibited [^3^H]DA uptake two-fold more in lesioned striatum and inhibited NE uptake ∼five-fold more than DA uptake in naïve striatum. Consequently, DA uptake may be mediated by NET when DAT loss is at PD levels. Increased inhibition of DA uptake by L-DOPA and its preferential inhibition of NE over DA uptake, indicates that NET-mediated DA uptake may be modulated by L-DOPA when DAT loss exceeds 70%. These results indicate a novel mechanism for DA uptake during PD progression and provide new insight into how L-DOPA affects DA uptake, revealing possible mechanisms of its therapeutic and side effect potential.

## Introduction

In striatum, the dopamine transporter (DAT) is a vital component for maintaining sufficient dopamine (DA) levels for release [1], [2]. Thus the degree of striatal DAT loss in Parkinson’s disease (PD) when locomotor symptoms appear (∼70–80%) [3], [4] would be expected to be a major factor in the deficit in DA that produces locomotor impairment. During the loss of DA-regulating proteins in PD progression, there is evidence that compensatory changes in DA regulation [5]–[7] may delay symptom presentation. For example, loss of DAT is concomitant with diminished DA release, which would be expected to sustain extracellular DA concentrations [8]. Increased TH activity may also maintain sufficient DA for some time during TH loss [5], [9], [10]. However, it is possible that DAT activity, like TH activity, could increase as a compensation mechanism to maintain cytosolic DA during DAT loss. Thus, the resulting increase in DA reuptake could diminish extracellular DA availability, thereby reducing synaptic concentrations necessary to bind post-synaptic DA receptors and drive locomotor activity. From the therapeutic perspective, it has been proposed that despite DAT loss, the efficacy of L-DOPA is first via its transport through other monoamine transporters. However, an overactive DA clearance mechanism, through remaining DAT, could conceivably also facilitate the transport of therapeutically-derived L-DOPA to produce DA via aromatic acid decarboxylase (AADC). Therefore, determining DA uptake dynamics when DAT loss is at and beyond the loss associated with locomotor symptoms is critical to understand the longevity of synaptic DA and the impact of L-DOPA in this context.

DAT function can regulate locomotor activity. DAT knockout mice exhibit hyperkinetic locomotor activity [11] and DAT blockade increases locomotor activity [12]. DAT levels are associated with DA turnover in the PD patient, implying that DAT plays an important role in maintaining DA bioavailability [13]. In advanced Parkinsonian monkeys and in PD patients, DAT function may be altered by the disease, but other monoamine transporters could also participate in DA uptake. For example, DAT inhibitors, particularly those with high norepinephrine transporter (NET)-, but low serotonin transporter (SERT)-affinity, provide increased locomotor benefits to monkeys with severe DAT loss (80%) compared with those with moderate DAT loss (46% loss) [14]. Serotonergic projections from the midbrain raphe nuclei to the striatum may regulate DA through the conversion of L-DOPA to DA in animals with 6-OHDA lesions [15], [16]. Still, despite DAT protein loss, the activity of remaining DAT may increase during PD progression to maintain intracellular DA levels in the face of decreased DA synthesis and storage capacity, due to loss of TH and VMAT2. Indeed, elimination of DAT by gene knockout drastically reduces DA tissue content in striatum [1]. Thus, DAT blockade may prove beneficial in PD patients. For instance, methylphenidate may provide modest improvement in locomotor deficiency in combination with L-DOPA [17]. Striatal DAT loss correlates with the less motorically-affected side of PD patients [18] suggesting that the more degenerated hemisphere has compensatory functions occurring that may affect accurate determination of DAT loss. Together, these data suggest the possibility of an overactive DA clearance mechanism in the nigrostriatal pathway when DAT protein loss reaches 80%that could diminish the synaptic DA levels that are required to drive locomotion.

Other monoamine transporters can transport DA in the CNS, particularly when DAT abundance is relatively low, as would be the case when locomotor symptoms present in PD. Although NET does not play a primary role in the clearance of DA in normal striatum, DA uptake occurs through NET in sparsely dopaminergic innervated regions such as the frontal cortex [19]. Moreover, selective NE uptake inhibitors can increase extracellular DA levels within the prefrontal cortex [20]–[25]. Conceptually, it is feasible that, NET or a NE-sensitive transport mechanism could potentially contribute to the clearance of DA in the DAT-impoverished Parkinsonian striatum, given that there is noradrenergic innervation of the striatum.

In a therapeutic context, there is the possibility that an overactive DA clearance mechanism could be a conduit for L-DOPA delivery into the aromatic acid decarboxylase (AADC)-expressing cells in the CNS, particularly since L-DOPA does not possess locomotor-enhancing properties until the threshold of loss occurs at ∼70–80% [26], [27]. Cell cultures expressing functional NET and DAT transport L-DOPA when it is present in high concentrations [28]. Systemic administration of the selective NET inhibitor desipramine increases extracellular DA derived from L-DOPA in 6-hydroxydopamine (6-OHDA)-lesioned rats, indicating that NET could play a significant role in DA clearance in the PD-like striatum and, consequently, may be involved in L-DOPA-derived DA synthesis in PD pathogenesis [29].

We determined differences in DA uptake in crude synaptosomes prepared from the 6-OHDA lesioned striatum versus inherently-matched contralateral intact striatum to determine the relationship of DAT loss to DA transport differences and potential involvement of monoamine transporters in lesioned terminals. We examined the extent by which 5-HT, NE, DA, (representing the endogenous monoamines) and L-DOPA (representing the gold-standard for PD treatment), affected [^3^H]DA uptake and also determined NET expression and impact of its inhibition on NE uptake to elucidate potential mechanisms by which DA is removed from the synapse with DAT loss at PD symptom levels, with and without L-DOPA present.

## Materials and Methods

### Animals

Male Sprague Dawley rats purchased from Harlan were used in all experiments. All rats were 4–8 months old in the study, and were housed under controlled lighting conditions (12∶12 light:dark cycle) with food and water available *ad libitum*. All animals were used in compliance with federal and the institutional Animal Care and Use Committee guidelines at LSU Health Sciences Center-Shreveport.

### 6-OHDA Lesions

Each animal underwent survival surgery to deliver the neurotoxin 6-OHDA to the medial forebrain bundle. Rats were anesthetized with 40 mg/kg Nembutal intraperitoneal (i.p.) (pentobarbital Lundbeck Inc, Deerfield, IL) with supplement of 9.0, 0.6, and 0.3 mg/kg ketamine, xylazine, and acepromazine, respectively. Animals were immobilized in a stereotaxic frame to target the medial forebrain bundle at coordinates ML +1.5, AP −3.8, DV −8.0 relative to Bregma according to Paxinos and Watson rat brain atlas, 4^th^ ed. [30]. A total of 9 or 16 µg of 6-OHDA in a total of 4 µl in 0.02% ascorbic acid (concentrations of 2.25 or 4 mg/ml) was infused unilaterally at a rate of 1 µl/minute. Notwithstanding possible bilateral effects of the 6-OHDA infusion, the contralateral striatum was left intact as a naïve tissue control. The syringe was left in place for 10 min before removal to allow for maximal diffusion of drug and to avoid further mechanical damage to the tissue. Body temperature was maintained at 37° during surgery using a temperature monitor with probe and heating pad (FHC, Bowdoingham, ME).

### Amphetamine Testing for Lesion Verification

Lesions were confirmed with amphetamine-induced rotation ipsilateral to the lesioned side. Rotational behavior was monitored for 60 minutes after a single i.p. injection of amphetamine (2 mg/kg) 7 days post 6-OHDA infusion. While the amphetamine-induced rotation is not as precise as apomorphine to detect lesion at 90% [31], we employed the amphetamine-induced rotation to be able to detect at least 50% lesion. Rats were sacrificed for DA uptake analysis and subsequent DAT or TH analyses 2 days after the amphetamine test to allow for near-complete clearance of amphetamine.

### Preparation of Synaptosomes

Synaptosomes were prepared according to the protocol previously described [32] with the following modifications: Tissue dissected from dorsal striatum and substantia nigra was homogenized in 5 mL of 0.32 M sucrose solution using a Teflon/glass homogenizing wand (Glas-Col, Terre Haute, IN) then spun at 1000×*g* for 10 minutes in a chilled (4°C) centrifuge. The resulting pellet was stored as the P1 fraction while the supernatant was spun further at 16,500×*g* for 30 minutes at 4°C, yielding the P2 fraction. An aliquot of the P1 fraction was saved for determination of TH protein from the 6-OHDA-lesioned and contralateral (control) striatum against a standard curve of TH protein standard [33]. The supernatant was aspirated and resuspended in 1 mL of Kreb’s buffer (118 mM NaCl, 4.7 mM KCl, 1.2 mM KH2PO4, 25 mM NaHCO3, 1.0 mM Na2EDTA, 1.7 mM CaCl2, 10 mM glucose, 100 µM parglyline, 100 µM ascorbic acid). Protein concentration was determined using a BCA colormetric assay (Thermo Scientific, Rockford, IL). All tissue was kept on ice or at 4°C from the moment of brain excision until the uptake assay took place.

### [^3^H]DA and [^3^H]NE Uptake into Synaptosomes

Synaptosomes were distributed in ice-cold test tubes to prepare for dopamine uptake. Given sufficient yield on protein recovery from the tissue for uptake, an aliquot of synaptosomes was also saved for later determination of the protein quantities of DAT. The determination of [^3^H]DA uptake in the crude synaptosomes from dorsal striatum harvested from the contralateral and 6-OHDA-infused hemispheres was conducted simultaneously and included assessments of uptake capacity in the presence of unlabeled 1 µM NE versus 1 µM DA, and 1 µM 5-HT or 1 µM L-DOPA. Each determination was done in triplicate for each assay condition and uptake was determined comparing the lesioned striatum with the contralateral control striatum. Non-specific uptake was determined by counts obtained in synaptosomes incubated with 500 nM DA (all as labeled DA) on ice during the time period of uptake. The determination of [^3^H]NE uptake in the crude synaptosomes from dorsal striatum harvested from the contralateral and 6-OHDA-infused hemispheres was conducted simultaneously at a final [NE] of 250 nM (all as labeled NE). Background was determined and subtracted in the same manner as in the DA uptake studies.

Synaptosomes (30 µg protein per replicate) were added to 4°C oxygenated Kreb's buffer and test ligand (if indicated) to reach a total volume of 100 µL volume. The synaptosomes were then warmed to 35°C for 5 min, then 100 µL of pre-warmed 1 µM ^3^H-dopamine, prepared from one of two sources of labeled DA; 1) ViTrax, [7-, 8- ^3^H-DA], specific activity of 25 Ci/mmol or 2) Amersham, [7-, 8- ^3^H-DA], specific activity 47 Ci/mmol, was added to the synaptosome preparations (giving a 500 nM final [^3^H]DA concentration), allowed to incubate for uptake, and terminated after 120 seconds with an excess volume of ice-cold Kreb’s buffer and re-immersing the tubes in the ice-bath. The uptake time for DA was chosen to be as close as technically and practically possible to the approximately 2-minute uptake time of striatal dopamine observed *in vivo* [34]. Labeled NE was purchased from Perkin-Elmer (levo- [7-^3^H]-norepinephrine; specific activity 14 Ci/mmol). We also conducted NE uptake for 2 minutes. Synaptosomes were washed extensively to remove excess labeled-dopamine with equal-osmolarity PBS buffer through a Brandel M24-TI (Gaithersburg, MD) cell harvester using Brandel GF/C filter paper pretreated with a 2% polyethylenimine solution to reduce non-specific binding of label. The filter paper containing the rinsed synaptosomes was transferred into scintillation vials containing 5 mL of biodegradable scintillation cocktail (Research Products International, Mount Prospect, IL) and counted with a Beckman Coulter LS6500 scintillation counter (Brea, CA).

### Calculating DA and NE Uptake

To determine the quantity of DA uptake, the percent of [^3^H]DA recovered in the synaptosomes against the total amount of [^3^H]DA added during the uptake experiment was first determined. This percentage of total labeled DA in solution taken up by the synaptosomes prepared from dorsal striatum averaged 0.55±0.07% (mean ±SEM, range 0.15 to 1.88%, *n = *27 experiments) using the Amersham [^3^H] DA and 1.63±0.18% (range 0.71 to 3.44%, *n = *18 experiments) using the ViTrax [^3^H]DA. Specificity of DA uptake was verified using cold DA as an inhibitor to uptake of labeled DA. The total pmole of recovered [^3^H]DA was then determined based upon this percent [^3^H]DA recovery in the synaptosomes after subtracting the non-specific binding value, and the result was normalized to synaptosome protein and expressed as pmole DA per mg protein per minute. The determination of NE uptake quantity was done in same way as DA uptake. The percentage of total [^3^H] NE in solution taken up in synaptosomes from dorsal stratum averaged 0.4% (*n* = 4 experiments).

### Tissue Preparation and Western Immunoblotting

Synaptosome pellets (to determine DAT protein, when available, ∼70% of experiments) and the processed preparatory sample (for TH protein assessment) were sonicated in a 1% sodium dodecyl sulfate solution (pH ∼8) using a Branson Sonifier 150 (Danbury, CT). Protein concentration was determined using the bichinchoninic acid colometric assay. Following gel electrophoresis, proteins were transferred for 500 volt hours in a Tris/glycine/methanol buffer onto nitrocellulose membranes (Bio-Rad Laboratories, Hercules, CA).

The nitrocellulose membrane was stained with Ponceau S to reveal relative protein staining in each sample lane. These lanes were scanned and quantified by Image J to normalize protein in each sample. This relative total level then served as an additional normalizing value to determine the quantity of each protein assayed [33]. To continue processing, the membranes were blocked in PVP buffer (1% polyvinylpyrrolidone and 0.05% Tween 20) for a minimum of two hours to reduce nonspecific antibody binding. The membrane was soaked in primary antibody for 1–3 hours. Specific primary antibodies were as follows: DAT (Santa Cruz, cat # sc-1433, 2 µg/ml), TH (Millipore, cat # AB152), and NET (Alpha Diagnostics Intl., cat# NET11-A). Protein loads for linear detection were 30 µg total protein for DAT and TH on the lesioned side, and 10 µg on the contralateral control side. Protein loads for NET were 60 ug in both lesioned and contralateral control side. After primary treatment, blots were exposed to secondary antibody (swine anti-rabbit IgG for TH and NET, swing-anti-goat IgG for DAT) for signal enhancement, followed by 1 h incubation with [^125^I] protein A (PerkinElmer, Waltham, MA).

### Statistics

All dopamine and norepinephrine uptake studies were done in conjunction with assessment of TH loss, and when possible, DAT loss, as assessed in aliquots of synaptosomes that were used to determine DA and NE uptake. Tissue harvested from the striatum contralateral to 6-OHDA-lesion served as the inherent control to the lesioned striatum for each rat/test subject. Therefore, a Student’s paired t-test was used to compare DA and NE uptake between the two sides, as well as to ascertain the degree of TH and DAT loss caused by 6-OHDA lesion. With the exception of comparing DA uptake (as per equal synaptosomal protein) between the two striata, the paired t-test was two-tailed. Given the expectation that there would be a decrease in DA uptake caused by the lesion, in the instance of comparing uptake as per equal protein, a one-tailed paired t-test was used.

## Results

### Dopamine Uptake in Non-lesioned Tissue and Consistency with Endogenous DAT

**Figure 1:**
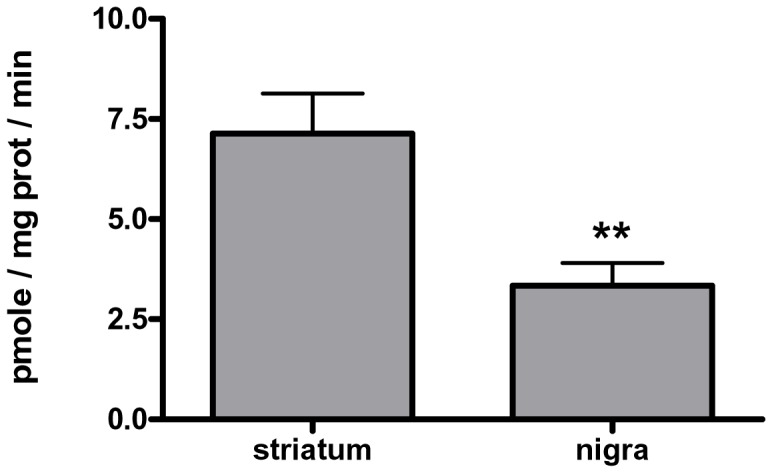
Dopamine uptake between striatum and substantia nigra. The inherent differences in DA uptake between striatum and substantia nigra in non-lesioned tissue, as per our synaptosome preparation and uptake protocol are illustrated. Our results reflect the previous observations that DAT expression is significantly less in SN than in striatum and *in vivo* assessments of DA clearance also show less DA uptake in the SN compared to striatum. Statistics, **p = 0.001, two-tailed paired t-test of 16 matched observations in synaptosomes prepared from striatum and substantia nigra dissected contralateral to medial forebrain bundle 6-OHDA lesion.

We first established that dopamine uptake in control tissue reflected the endogenous quantities of transporter, wherein there is greater DA uptake [35]–[37] and DAT protein [38] in dorsal striatum versus substantia nigra. In the control non-lesioned tissue, DA uptake was significantly greater in synaptosomes from the striatum than from the substantia nigra (SN) (Figure 1). This difference is in agreement with previous findings [35]–[37].

### Dopamine Uptake in Relation to Loss of Tyrosine Hydroxylase

To verify the degree of lesion in association with DA uptake in the lesioned versus contralateral control tissue, we determined TH loss using tissue not utilized for the synaptosome fraction for reuptake studies in all test subjects. When tissue recovery in the synaptosome fraction was adequate to do so, we also determined DAT loss in aliquots to normalize DA uptake to the loss of DAT. There was a significant correlation of TH to DAT loss, ranging from 61 to 99% loss (9 observations, Pearson r = 0.921, *p* = 0.0004, two-tailed; data not shown), so the degree of TH loss, when not possible to determine DAT loss, reflected DAT loss.

**Figure 2:**
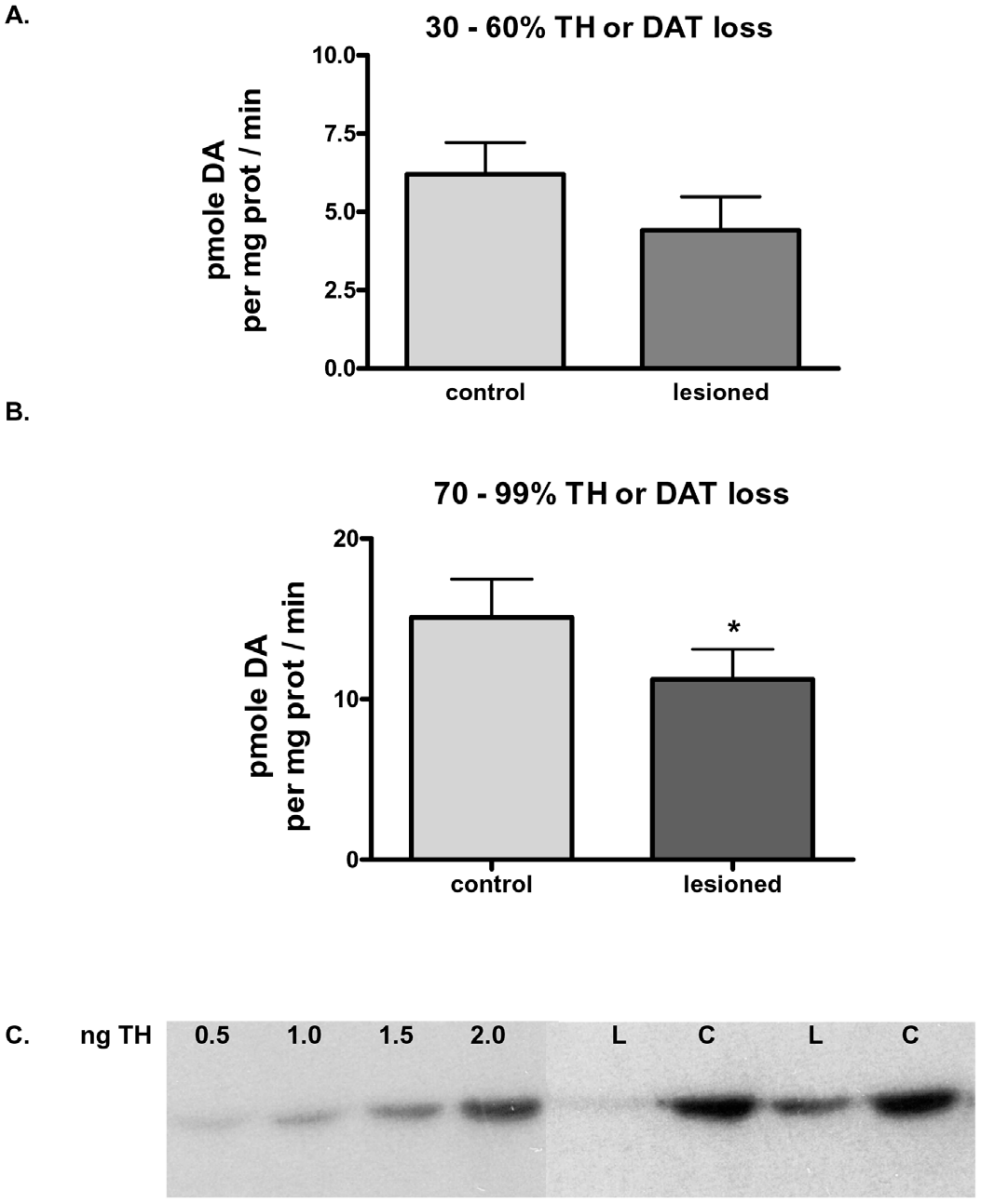
Dopamine uptake profiles per equal synaptosomal protein related to percent loss of tyrosine hydroxylase. A. DA uptake with TH loss at 30–60%. Statistics, *p = *0.055, one-tailed paired t-test of 6 matched observations in synaptosomes prepared from striatum ∼9 days following mfb 6-OHDA lesion. TH loss was confirmed in a tissue fraction during synaptosome preparation. **B.** DA uptake with TH loss at 30–60% at 70–99% loss. Statistics, *p*<0.05, one-tailed paired t-test of 26 matched observations in synaptosomes prepared from striatum ∼9 days following mfb 6-OHDA lesion. TH loss was confirmed in a tissue fraction during synaptosome preparation. **C.** Representative western blot depicting TH loss. TH loss by the 6-OHDA lesion **(L)** is shown versus quantity in contralateral striatum **(C)** and interpolation by accompanying standard curve of TH protein (0.5 to 2.0 ng TH). Associated Ponceau stain (below TH bands) on same blot before TH antibody blotting demonstrates similar striatal protein loading.

As our assay revealed differences in DA uptake based upon inherent DAT levels (Fig. 1), we found an unexpected result in DA uptake in the verified lesioned neuropil. We expected to observe a significant decrease in DA uptake in the lesioned neuropil. However, there was only a trend toward a decrease in DA uptake in rats with confirmed lesion varying between 30 to 60% loss (Fig. 2A). Even more striking was that while there was a significant decrease in DA uptake in rats with at least 70% loss, the magnitude of TH or DAT loss was much greater than the reduction in DA uptake of ∼26% (Fig. 2B, 2C). These findings reveal the possibility that remaining DAT protein could have greatly increased DA uptake capabilities or that another monoamine transporter is active in the lesioned striatum for DA uptake.

### Role of DAT in 6-OHDA Lesioned Striatal Uptake

**Figure 3:**
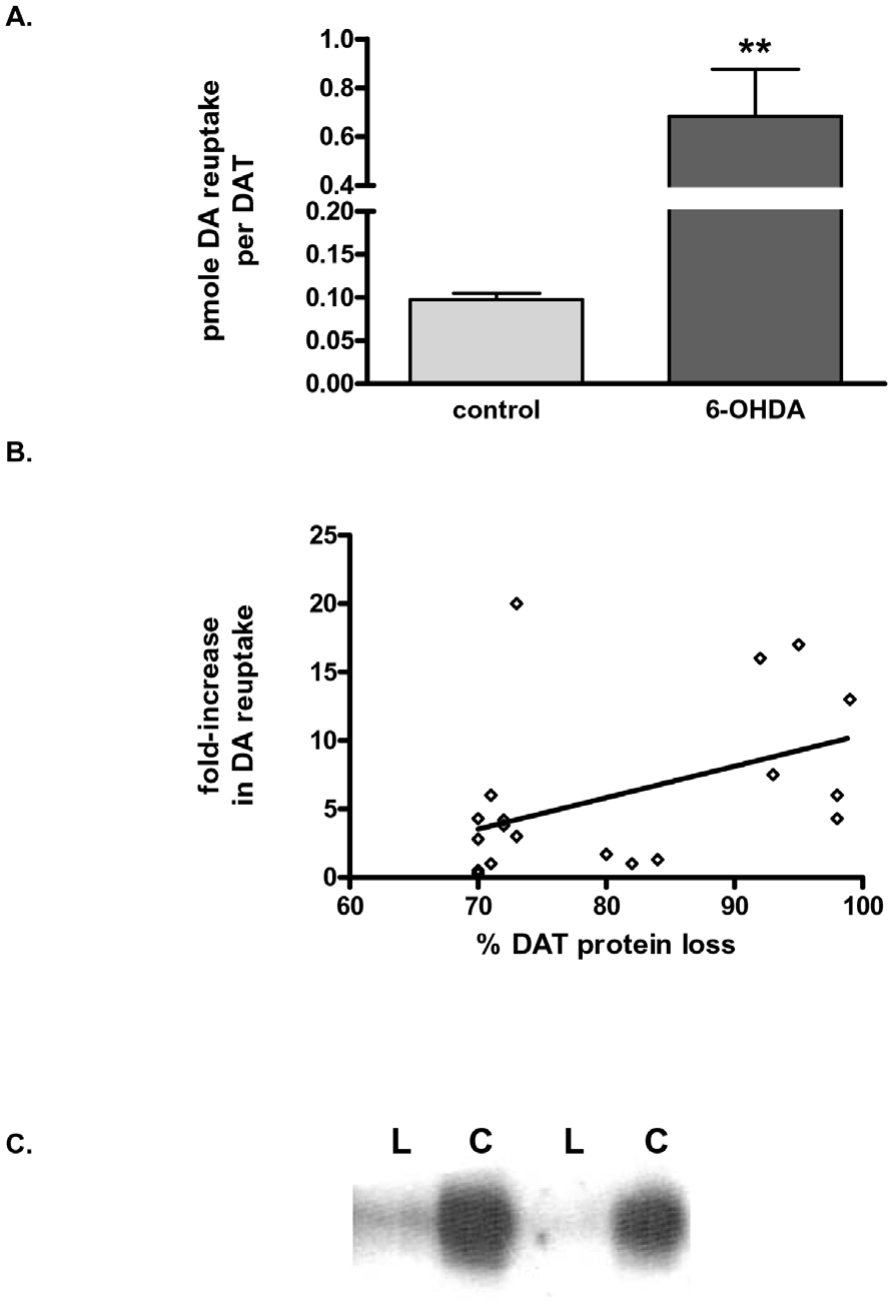
Dopamine uptake per remaining DAT protein. A. DA uptake in synaptosomes normalized to remaining DAT protein recovered in the synaptosome fraction with confirmed DAT loss of at least 70%. Statistics, **p<0.01, t = 3.49, two-tailed paired t-test of 19 matched observations in synaptosomes prepared from striatum ∼9 days following mfb 6-OHDA lesion. **B.** Significant correlation of DA uptake with the severity of DAT loss. Statistics, *p* = 0.015, Spearman correlation analysis of DA uptake versus %DAT protein loss, r = 0.55, *n = *19 pairs. **C**. Inherent DAT immmunoreactivity in synaptosome aliquots of equivalent total protein quantity from the 6-OHDA-lesioned (L) and contralateral striatum (C).

When we normalized DA uptake to the respective DAT protein at ≥70% DAT loss, DA uptake per DAT protein remaining was increased ∼6-fold (Fig. 3A). There was also a significant relationship in DA uptake with lesion progression, in that as the lesion severity increased, so did the DA uptake as per remaining DAT protein (Figure 3B,C). These results partially explain why DA uptake in the lesioned synaptosomes does not decrease in concert with DAT protein loss, as shown in figure 2B and suggests that another monoamine transporter may be more active in DA uptake under these conditions.

### Monoamine Inhibition of DA Uptake in Lesioned Striatum

**Figure 4:**
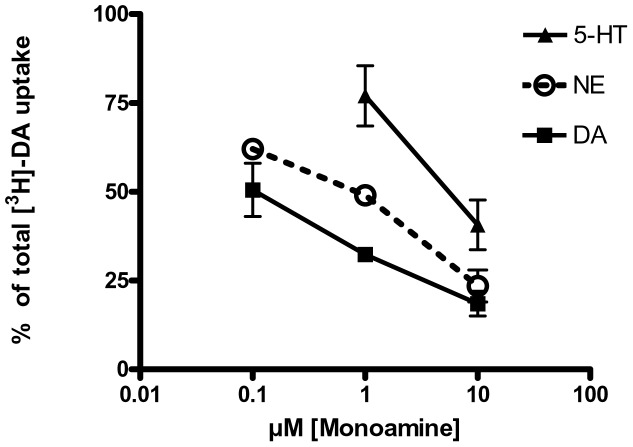
Establishment of uptake competition differences between the endogenous monoamines of striatum in naïve tissue. The percentage of DA uptake was assessed in the presence of varying concentrations of NE, DA and 5HT in the naïve striatum. The concentration of 1 uM of NE, DA and 5HT differentially inhibited uptake of labeled DA, and was thus chosen for discerning involvement of other monoamine transporters in the paradoxical increase of DA uptake per remaining DAT (as seen in Fig. 3).

The 6-fold increase in DA uptake per remaining DAT protein indicates the possibility that in lesioned striatum, DAT affinity for DA increases or another monoamine transporter could have involvement in DA uptake. To investigate these possibilities, we determined the relative Ki of the monoamines endogenous to striatum, between DA, NE, and 5-HT, and determined the efficacy of the unlabeled monoamines to inhibit [^3^H]-DA uptake in our striatal synaptosome preparation. As expected, DA was most effective at inhibiting DA uptake in naïve striatum, followed by NE, and serotonin (5-HT) (Fig. 4). With regard to the relative affinities of DA versus NE, our finding was supported by previous work [39], wherein K_m_ for DA and NE in striatum were ∼400 nM and 2 µM, respectively, and suggests that DAT affinity for DA is greater than NE in intact striatum. Full kinetics were not performed in lesioned rats due to limited availability of dissected intact striatal tissue, thus hindering the execution of complete pharmacokinetic profiles. However by performing this experiment in intact striatal tissue, we were able to determine that a concentration of unlabeled monoamine of 1 µM inhibited DA uptake to different and discernable degrees and thus help to discern the potential involvement of other monoamine transporters in DA reuptake in the lesioned striatum.

**Figure 5:**
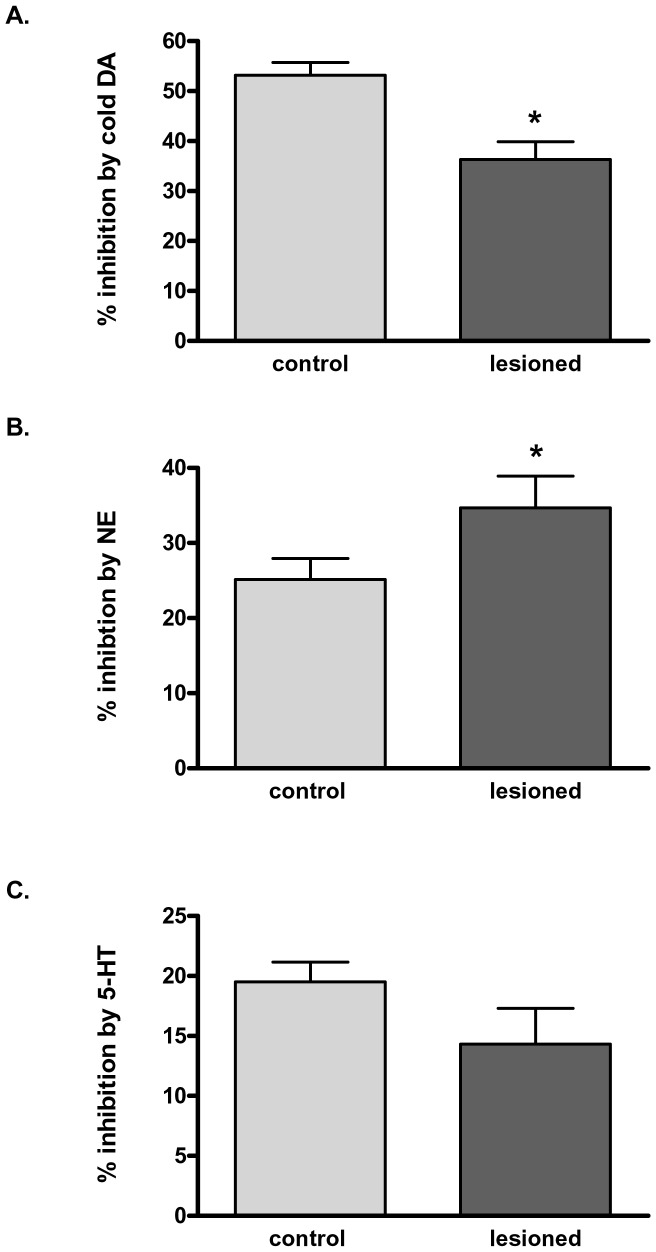
Dopamine uptake profiles with monoamine competition in 6-OHDA-lesioned striatum versus intact striatum. A. Dopamine. 1 µM DA was added in striatal synaptosomes prepared from at least 70% lesioned striatum and from the operationally-matched contralateral control. After 5 min preincubation period, 500 nM [7-, 8- ^3^H-DA] was added and uptake was determined for 2 min. In the lesioned striatal synaptosomes, DA was significantly less effective (30% less inhibition than in control) effective to inhibit DA uptake, as compared to the control. Statistics: *p<0.05, t = 3.47, two-tailed Student’s paired t-test, *n = *6 paired observations. **B. N**orepinephrine 1 µM NE was added in striatal synaptosomes prepared from at least 70% lesioned striatum and from the operationally-matched contralateral control. After 5 min preincubation period, 500 nM [7-, 8- ^3^H-DA] was added and uptake was determined for 2 min. In the lesioned striatal synaptosomes, NE was significantly more effective (38% greater inhibition than in control) to inhibit DA uptake, as compared to the control. Statistics: *p<0.05, t = 2.59, two-tailed Student’s paired t-test, *n = *6 paired observations. **C.** Serotonin 1 µM 5-HT was added in striatal synaptosomes prepared from at least 70% lesioned striatum and from the operationally-matched contralateral control. After 5 min preincubation period, 500 nM [7-, 8- ^3^H-DA] was added and uptake was determined for 2 min. There was no significant difference in the ability of 5-HT to inhibit DA uptake in lesioned striatal synaptosomes, as compared to the control, *n = *6 paired observations.

To determine if remaining DAT had increased affinity for DA in the >70% lesioned striatum, 1 µM unlabeled DA was used to compete with uptake of [^3^H]DA (500 nM). Compared to 53% inhibition in the contralateral control striatal synaptosome, the inhibition of [^3^H]DA uptake by unlabeled DA (1 µM) was significantly reduced in lesioned striatum to 34% (Fig. 5A). Therefore, increased affinity for DA may not play a role in enhanced DA uptake after ≥70% loss of DAT protein. However, in simultaneously-run uptake experiments derived from synaptosomes prepared from the same 6-OHDA-lesioned rat, NE, at an equal concentration to DA, inhibited DA uptake to a 40% greater extent in synaptosomes from the lesioned striatum (Fig. 5B). Given that others have found that NET is involved in DA uptake in DAT-impoverished regions of brain [19], [24], these results indicate that NET or another NE-sensitive transporter could mediate DA uptake in dopaminergic neuropil when loss of DAT exceeds 70%.

It has been shown that SERT binding is decreased in PD patients [40] which would argue that one possible route of L-DOPA uptake into an AADC source is diminished. In order to investigate the contribution of SERT in DA uptake, we also examined the ability of 1 µM of 5-HT to inhibit [^3^H]-DA uptake. There was no significant difference in the ability of 1 µM 5-HT to inhibit [^3^H]DA uptake in lesioned compared to control striatum (Fig. 5C).

### Impact of 6-OHDA Lesion on NET Expression and NE Uptake

**Figure 6:**
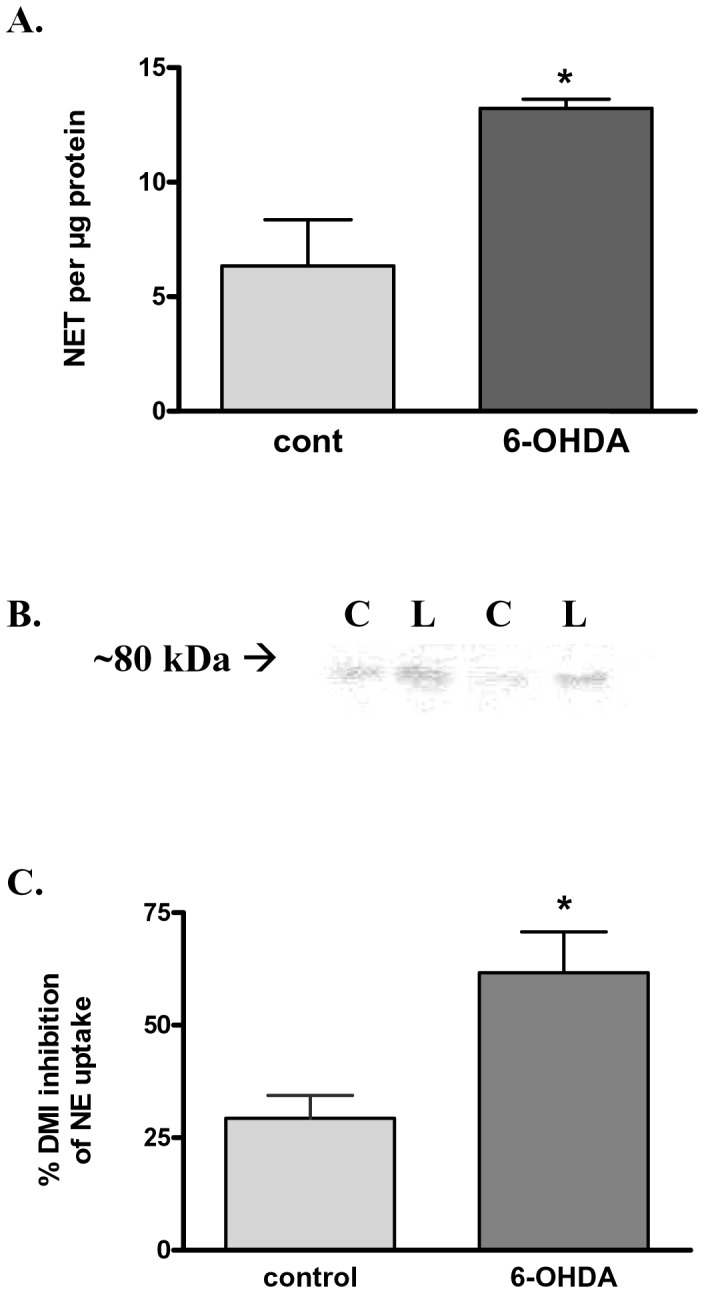
Norepinephrine Transporter (NET) striatal protein expression and function in 6-OHDA lesioned rats at >70% TH protein loss. A . NET protein expression. Quantification of NET showed that NET was significantly increased in lesioned striatum compared to its matched non-lesioned control. Statsitics: NET, *p<.05, t = 3.251, n = 4 paired observations. **B**. Representative western blot of NET expression. Relative expression of NET (∼80 kDa band) between contralateral, control, striatum (**C**) and 6-OHDA-lesioned striatum (**L**). **C**. Increased NET function in 6-OHDA lesioned striatum. Desipramine (DMI)-mediated inhibition of [^3^H] NE uptake between contralateral (control) and lesioned striatum (mean TH loss = 66%). Statistics *p*<0.05, t = 3.127, *n* = 3 observations.

In separate studies, we examined the impact of our 6-OHDA protocol on striatal NET expression and function, as well as monoamine tissue content in the same tissue sources to determine if the lesion impacted NE or 5-HT terminals. We observed that NET protein expression significantly increased in the 6-OHDA lesioned neuropil with >70% loss of TH (Fig. 6A, B). Desipramine, a NET-specific inhibitor, inhibited NE uptake to a significantly greater extent in lesioned striatum (Fig. 6C).

**Figure 7:**
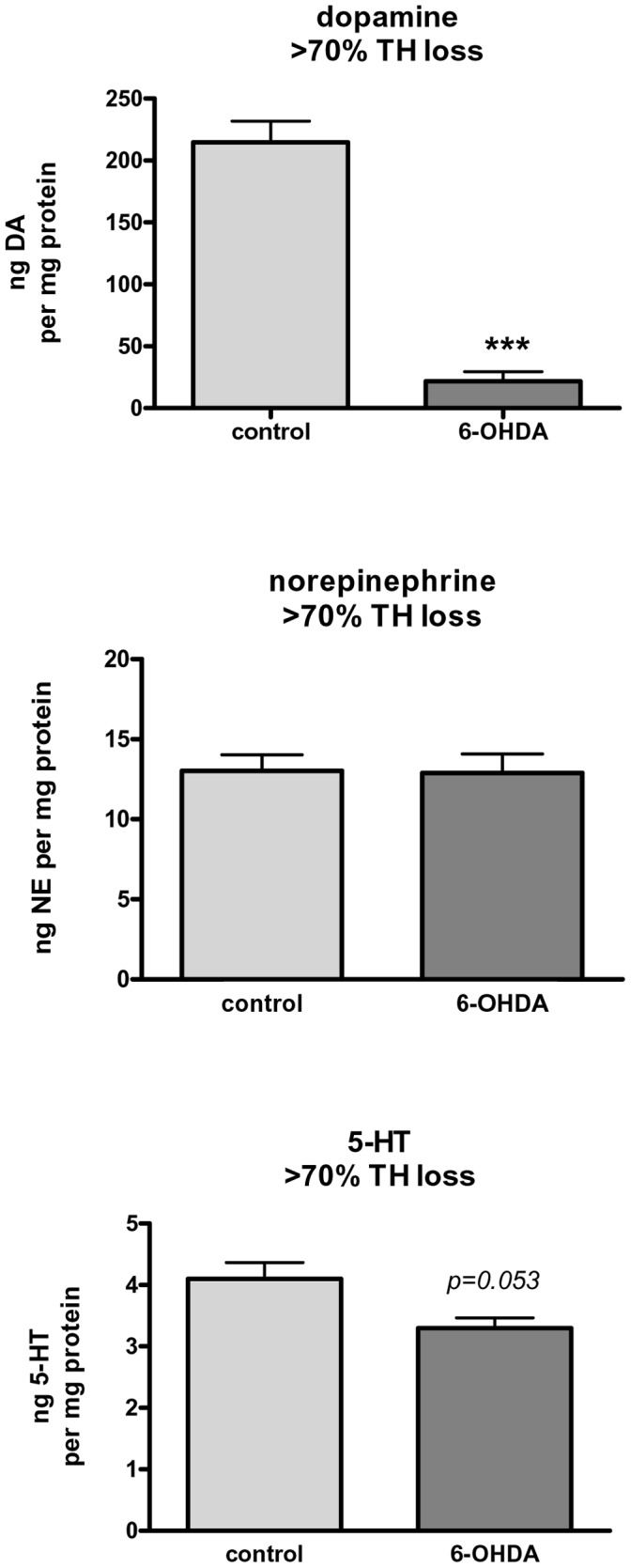
Monoamine content in operationally matched 6-OHDA lesioned rats at >70% TH protein loss. Representation of relative impact of our 6-OHDA lesion protocol on monoamine tissue content (expressed as ng monoamine per mg protein) in striatum with at least 70% confirmed loss of dopamine (top panel). As shown in the middle panel, there was no loss of NE in the 6-OHDA lesion method employed, but there was a trend toward a decrease in 5-HT (bottom panel). Statistics: DA, ***p<0.0001, t = 10.87. *n* = 7 paired observations for all monoamines.

In the tissues wherein we determined NET protein expression, we also determined relative monoamine tissue content by HPLC using a protocol that can analyze monoamine content and recovered proteins from the same sample [33]. In the intact, non-lesioned striatum, relative monoamine tissue content (per mg protein) was predictably dominated by DA (215±17 ng), then NE (13.0 ng), and 5-HT (4.1 ng). Given at least 70% loss of DA caused by our lesion, our lesion produced no significant effect on NE tissue content (Fig. 7). Serotonin tissue content, which was significantly less than NE tissue content in striatum, was not significantly affected, although there was a notable trend toward a decrease (*p = *0.055). Given that our lesion protocol did not reduce NE tissue content, we speculate NE-terminal proteins, such as NET, were not likely affected by the lesion. However, the increase in NET expression, despite no loss of NE tissue content, suggests that increased NET expression may be from a non-neuronal source.

### Impact of L-DOPA on DA Uptake

**Figure 8:**
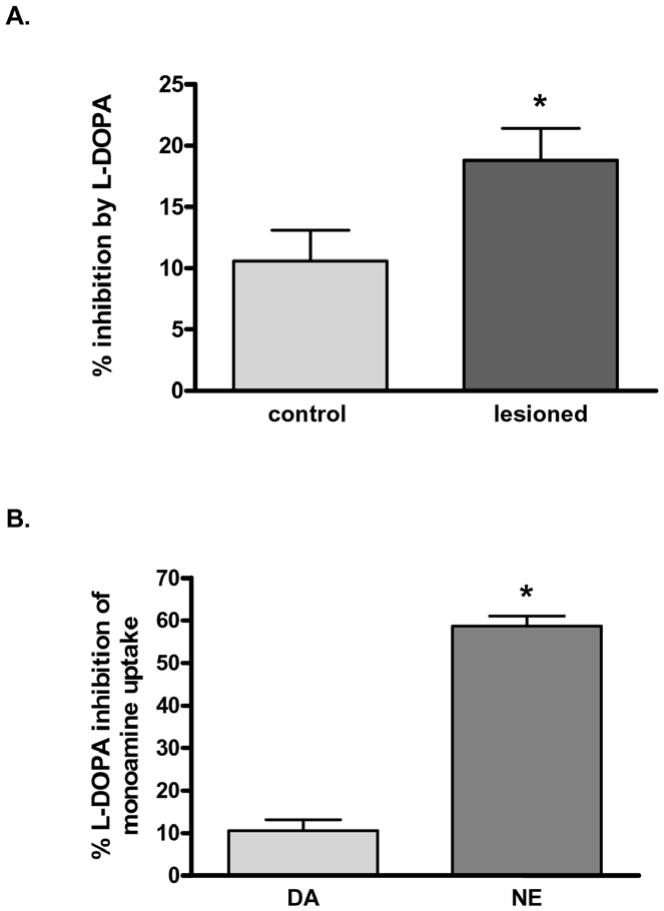
Impact of L-DOPA on monoamine uptake A. Dopamine uptake in lesioned striatum in presence of L-DOPA. 1 µM L-DOPA was added in striatal synaptosomes prepared from at least 70% lesioned striatum and from the operationally-matched contralateral control. After 5 min preincubation period, 500 nM [7-, 8- ^3^H-DA] was added and uptake was determined for 2 min. In the lesioned striatal synaptosomes, L-DOPA was significantly more (77% above inhibition in control) effective to inhibit DA uptake. Statistics: *p<0.05, t = 3.31, two-tailed Student’s paired t-test, *n = *5 paired observations. **B.** Impact of L-DOPA on DA versus NE uptake. 1 µM L-DOPA was added to naïve (unlesioned) striatal synaptosomes and after 5 min preincubation, either 500 nM ^3^H-DA or 250 nM [^3^H] NE was added and uptake was determined for 2 min. Statistics (*p<0.001, t = 12.75, unpaired two-tailed Student’s t-test, *n* = 4 for NE, 5 for DA).

The primary pharmacological treatment for patients with PD is L-DOPA, the biosynthetic product of TH. Aromatic acid decarboxylase (AADC) immunoreactive cells have been identified in conjunction with presence of DA in denervated striatum following the administration of L-DOPA [41]. L-DOPA crosses the blood-brain barrier to reach the denervated nigrostriatal pathway in PD, but how L-DOPA is transported into AADC-expressing cells is not completely understood. We examined whether L-DOPA affected [^3^H]DA uptake differently in the 6-OHDA lesioned striatum versus intact striatum. L-DOPA (1 µM) was nearly twice as effective at blocking [^3^H]DA uptake in lesioned striatum (19% inhibition), compared to 11% in contralateral control striatum (Fig. 8A). This increased ability of L-DOPA to block [^3^H]DA uptake when DAT loss ≥70% support the idea that L-DOPA itself may extend the life of DA in the Parkinson’s synapse and its reuptake may be mediated by a transport mechanism distinct from the DAT, as suggested by results presented in Figures 5 and 6.

In naïve striatal tissue, L-DOPA was significantly more effective at inhibiting the uptake of NE compared to DA (Fig. 8B), which suggests that L-DOPA has a greater affinity for NET than DAT. This finding may also support the possibility that if NET is active in DA uptake in lesioned striatum, then it would be expected that L-DOPA would be more effective at inhibiting DA uptake, as indicated in Fig. 8A.

## Discussion

The results presented here contribute to the increasing body of literature that supports that synaptic DA levels are still regulated in DAT-impoverished regions of the CNS. This has been observed in the PD patient and in PD models [42]–[44]. Our study extends the observations that the function of remaining DAT changes with lesion severity by the notion that other monoamine transporters could contribute to the regulation of DA in the synapse [43], [45], [46]. In our hands, when >70% loss of DAT protein was produced by 6-OHDA, there was a paradoxical 6-fold increase in [^3^H]DA uptake per remaining DAT protein in the lesioned striatum. Similarly, Khakimova and colleagues [47] also observed increased [^3^H]DA uptake by the remaining nigrostriatal neurons (both in striatum and substantia nigra) in a mouse PD model at pre-symptomatic and early symptomatic stages. In conjunction with our finding that DA was less effective at blocking its own uptake in the lesioned versus intact striatum, it is possible that other monoamine transporters account for the enhanced [^3^H]DA uptake observed in this study. This possibility has implications for modifying therapies that target other monoamine transporters to improve the longevity of DA in the synapse. The ability of L-DOPA to inhibit DA uptake in lesioned striatum also has therapeutic implications. First, from the standpoint of its locomotor benefits, our results suggest the partial blockade of DA uptake by L-DOPA that occurs paired only with DAT loss associated with PD symptoms, may help to extend the longevity of DA in the synapse. However from the standpoint of L-DOPA-induced dyskinesia, a common side effect of chronic L-DOPA use, the results suggest that any L-DOPA in excess of what is needed for DA synthesis could impair DA reuptake and increase synaptic levels of DA, exacerbating DA receptor hypersensitivity seen in dyskinesia pathophysiology.

We acknowledge that our conclusion is incomplete from the perspective of clearly identifying the monoamine transporters involved with DA reuptake at the severe lesion stage. However, the combination of several independent results suggest a role for NET in DA uptake in PD progression. Blockade of [^3^H]DA uptake by NE was more effective in the lesioned striatum compared to contralateral control striatum, suggesting a NE-sensitive compensatory mechanism for DA uptake. Given the drastic loss of DAT observed with the 6-OHDA lesion and the decreased effectiveness of DA to block [^3^H]DA uptake in lesioned striatum, the most straightforward explanation is increased DA clearance by NET, which may transport DA with higher affinity than the DAT itself in some cases [48]–[50]. Support for a NET-mediated mechanism is further evidenced by NE uptake being inhibited by desipramine to a greater extent in 6-OHDA lesioned striatum (Fig. 6C). Additionally, the increase in NET protein levels seen with >70% TH loss (Fig. 6 A,B) also indicates that this mechanism may be compensatory thereby augmenting DA reuptake through NET when DAT is sparse.

Previous work gives some support to our results that NET-mediated DA uptake can occur when DAT levels are inherently low. Initial studies performed with cloned hNET expressed in transfected cells indicate that the NET has a greater affinity for DA than for NE [51]. Spatial differences in desipramine-sensitive DA clearance in the substantia nigra positively correlate with dopamine-β-hydroxylase in naïve brain slices, suggesting that the NET-mediated DA reuptake in some [35], but not other [52], regions are likely due to a much larger quantity of DAT. However, when the DAT protein is diminished, as with 6-OHDA, the primary route of DA uptake may be shifted to NET, or at least NE-sensitive transporters. For instance, NET-mediated DA uptake occurs when the DAT is genetically or pharmacologically inactivated [19] or in brain regions of low dopaminergic innervation like the prefrontal cortex or hippocampus [53]–[55]. Thus, our results could reflect how DA is regulated in the synapse with low DAT expression.

Our results were not supportive of, but did not eliminate the possibility, that 5-HT-sensitive mechanism may be at work for the paradoxical increase in DA uptake, given remaining DAT protein. The serotonin transporter (SERT) may transport both NE and DA, particularly at high DA concentrations [56]–[57]. However, SERT-mediated DA uptake was apparently not altered in the 6-OHDA lesioned striatum, at least at the DA concentration chosen (500 nM), because if SERT was more active in lesioned striatum, 5-HT would inhibit the accelerated DA uptake (Fig. 5C). This observation may at first seem at odds with previous studies, that suggest serotonin terminals convert L-DOPA to produce DA [58]–[59]. However, if L-DOPA is predominantly converted to DA in serotonin terminals, it is still conceivable that remaining L-DOPA could block uptake of extracellular DA, as indicated by our results. It is important to note, however, that SERT levels do decrease during PD progression [60]. Thus, our data indicates that NET also plays a role in DA clearance dynamics and the fate of L-DOPA, in addition to that previously demonstrated by DAT or SERT in PD progression.

Another alternative explanation for the observed NE-sensitive uptake of [^3^H]DA in lesioned striatum is transport activity from high-capacity but low-affinity transporters, such as the plasma membrane monoamine transporter (PMAT) or the organic cation transporters (OCTs). Uptake activity by the PMAT is sensitive to NE and DA, but is most sensitive to 5-HT [61]–[62]. The PMAT is likely insensitive to blockade of [^3^H]MPP^+^ uptake by L-DOPA [63]. Thus, at least the literature support the idea that it is unlikely that the PMAT is the NE-sensitive transport mechanism revealed in our study, because 5-HT was least effective at blocking [^3^H]-DA uptake compared to NE and DA. However, the OCT subtypes 2 and 3 have affinity for NE, DA and 5-HT [64]. The OCT3 binds these monoamines more effectively than the OCT2, with IC_50_ values being lowest for NE and over threefold greater for 5-HT [65]–[66]. Therefore, we cannot definitively rule out involvement of OCT3 in the observed NE-sensitive [^3^H]DA clearance in lesioned striatum and this possibility merits further examination.

Given that we did not observe any change in NE or 5-HT tissue content (Fig. 7), we presume that this would signify little change in proteins, like NET and SERT, expressed by these terminals. Therefore, it is logical to ask what cellular entity could contribute to increased DA uptake and NET expression in the lesioned striatum. One possibility is the glial cell. Astrocyte and microglia cell numbers may increase in PD as a part of the inflammatory response associated with the progressive loss of dopaminergic neurons (for review see [67]). Astrocytes may also regulate extracellular DA, as they functionally express DAT, NET, and OCT3 [68]–[70]. Astrocytes also express AADC, and may convert L-DOPA to DA [71]–[72] thus serving as a source of DA, via uptake of L-DOPA. Indeed, NET, but not SERT, blockers may inhibit both [^3^H]DA and [^3^H]NE uptake in astrocytes [68]. The increase in NET expression in the 6-OHDA-lesioned striatum, in conjunction with no increase in NE tissue content, may suggest that the cellular source of increased NET is from the astrocytes, rather than NE terminals that sparsely innervate the dorsal striatum. Therefore, it is possible that as PD progresses, increased numbers of astrocytes or microglia in striatum may provide an additional route of DA uptake or L-DOPA transport, which would either reduce synaptic DA available for neurotransmission or be a cellular entity that produces DA from exogenous L-DOPA.

### L-DOPA

The use of L-DOPA in the treatment of the PD patient remains the primary pharmacological tool to ameliorate locomotor dysfunction [73], and its efficacy lies, in part, in its ability to increase DA in the PD patient [74]. However, the question remains as to why L-DOPA is effective when the proteins involved with its handling, (1) DAT, which would transport exogenous L-DOPA into remaining DA neuropil, and (2) AADC, which would catalyze the conversion of L-DOPA to DA, are diminished to the same degree as TH [75]. Our data may provide some additional insight into how L-DOPA could benefit the PD patient, as we observed that it produced a nearly two-fold greater ability to inhibit [^3^H]DA uptake in the lesioned striatum over the intact striatum. It might even be possible that L-DOPA itself is subject to greater uptake in the striatum of PD patient. Either possibility lends itself to a therapeutic benefit from a first glance, notwithstanding complications of L-DOPA therapy, notably L-DOPA-induced dyskinesia [76]–[78] over long-term use. Given the propensity for chronic L-DOPA therapy in PD treatment to induce L-DOPA induced dyskinesia, the mechanism by which L-DOPA works, and ultimately fails, remains a clinically relevant issue. There is evidence of noradrenergic involvement in the pathogenesis of L-DOPA-induced dyskinesia [79]. In the striatum of the 6-OHDA lesioned rat, L-DOPA-derived DA is cleared from the extracellular space primarily by the NET [29]. This result is complemented by other work demonstrating that DAT blockade has no effect on DA that originates from L-DOPA in 6-OHDA lesioned striatum [15]. Very recent evidence also shows that alpha-synuclein, a protein that is implicated in PD pathogenesis, may interfere with DAT transport capabilities [80]. In line with these studies, and our data, a NE-sensitive transporter like NET could therefore be a clinically relevant therapeutic target in alleviating L-DOPA induced dyskinesia.

### Conclusions

Our results show that in spite of considerable loss of DAT, there remains a measurable quantity of DA uptake that is not diminished to the degree of DAT protein loss, and is preferentially inhibited by NE and L-DOPA. An increase in desipramine-mediated inhibition of NE uptake in conjunction with increased NET expression supports the possibility that DA uptake in lesioned striatum may be mediated, to a large degree, by NET. The preferential inhibition of DA uptake by L-DOPA in lesioned striatum suggests L-DOPA could enable extracellular DA to remain in the synapse for a longer period of time. However, that NE tissue content was not affected by our lesion, suggests that NET-mediated DA uptake may not be mediated by NE terminals, but another cellular source such as glia. This leads us to speculate that, given glia express NET and thus could represent an abundant source of NET in striatum, that DA could be regulated by more than the monoamine transporters expressed on monoamine terminals. Further investigation of the cellular and molecular mechanisms of NET-mediated DA reuptake when DAT loss is at and beyond the degree associated with PD-associated motor symptoms could prove beneficial for locomotor capabilities in addition to providing a potential therapeutic target in the treatment of L-DOPA induced dyskinesia.
